# Gender-Specific Differences in Diastolic Dysfunction and HFpEF: Pathophysiology, Diagnosis, and Therapeutic Strategies

**DOI:** 10.3390/jcdd12060213

**Published:** 2025-06-05

**Authors:** Francesca Coppi, Gianluca Pagnoni, Francesca Grossule, Ashraf Nassar, Arianna Maini, Giuseppe Masaracchia, Francesco Sbarra, Elisa Battigaglia, Enrico Maggio, Daniela Aschieri, Federica Moscucci, Marcello Pinti, Anna Vittoria Mattioli, Francesco Fedele, Susanna Sciomer

**Affiliations:** 1 Department of Medical and Surgical Sciences for Children and Adults, University of Modena and Reggio Emilia, Via del Pozzo 71, 41124 Modena, Italy; 2 National Institute for Cardiovascular Research (INRC), Via Irnerio 48, 40126 Bologna, Italy; 3 Cardiology Unit of Emergency Department, Guglielmo da Saliceto Hospital, 29121 Piacenza, Italy; 4 Cardiology Division, Department of Biomedical Metabolic and Neural Sciences, University of Modena and Reggio Emilia, Via del Pozzo 71, 41124 Modena, Italy; 5 Department of Clinical Internal, Anesthesiological and Cardiovascular Sciences, Sapienza University of Rome, 00185 Rome, Italy; 6 Dipartimento di Scienze Cliniche, “Sapienza” Università degli studi di Roma, 00185 Rome, Italy; 7 Department of Life Sciences, University of Modena and Reggio Emilia, Via G. Campi 287, 41125 Modena, Italy; 8 Department of Quality of Life, University of Bologna—Alma Mater Studiorum, 40126 Bologna, Italy; 9 Emeritus of Cardiology, Sapienza University of Rome, 00185 Rome, Italy; 10 National Institute for Cardiovascular Research (INRC), 40138 Bologna, Italy

**Keywords:** heart failure, diastolic dysfunction, HFpEF, echocardiography, gender differences, SGLT2 inhibitors

## Abstract

**:** Heart failure with preserved ejection fraction (HFpEF) accounts for approximately 50% of heart failure cases and is primarily characterized by impaired diastolic function, leading to increased ventricular filling pressures and symptoms like dyspnea and reduced exercise tolerance. Significant gender-specific differences are observed, with women, particularly post-menopausal, experiencing higher prevalence and distinct clinical profiles compared to men. Diastolic dysfunction in HFpEF involves altered cellular mechanisms such as reduced SERCA2a expression, impacting calcium handling and myocardial relaxation. Diagnostic strategies mainly employ echocardiography, including Doppler imaging, tissue Doppler imaging, and strain imaging, to assess ventricular relaxation and stiffness. However, early identification remains challenging, necessitating advanced tools like cardiac magnetic resonance and exercise stress testing for accurate diagnosis, especially in women who often present with subtle symptoms. Treatment options for HFpEF have traditionally been limited, but recent trials, notably EMPEROR-PRESERVED and DELIVER, demonstrated significant cardiovascular benefits using sodium–glucose cotransporter-2 (SGLT2) inhibitors. Additionally, glucagon-like peptide-1 receptor agonists (GLP-1 RAs) have shown promising results, particularly in obese patients. Despite these advances, gender differences in therapeutic response necessitate further research for personalized management strategies. Understanding sex-specific pathophysiological mechanisms and optimizing diagnostic criteria remain essential to improving prognosis and quality of life in HFpEF patients.

## 1. Introduction

Heart failure with preserved ejection fraction (HFpEF) accounts for approximately 50% of heart failure cases and is characterized by impaired diastolic function, leading to increased ventricular filling pressures and clinical symptoms such as dyspnea and exercise intolerance [1,2,3].

Diastolic dysfunction is driven by cellular and hemodynamic alterations, including delayed myocardial relaxation and increased ventricular wall stiffness. At the cellular level, relaxation occurs through the detachment of actin–myosin cross-bridges, regulated by calcium reuptake via the SERCA2a pump. In patients with HFpEF, reduced SERCA2a expression impairs ventricular relaxation [4].

From a diagnostic perspective, Doppler echocardiography is the primary tool for assessing diastolic function. One of the most significative studies on diastolic function using transthoracic echocardiography was published 43 years ago [5].

Subsequently, more refined diagnostic criteria were developed, culminating in the recommendations of the American Society of Echocardiography and the European Association of Cardiovascular Imaging [6].

Subsequent multicenter studies have confirmed the validity of these criteria in diagnosing HFpEF through invasive measurements of filling pressure [7,8].

Epidemiologically, HFpEF is more common in females during menopausal transition and during postmenopausal codified period, mainly due to increased left ventricular stiffness associated with differences in the extracellular matrix and the postmenopausal decline in estrogen levels, leading to altered vascular and metabolic function [9,10].

From a clinical perspective, diagnosis is often challenging due to nonspecific symptoms. Traditional echocardiography may not be sufficient, and the integration of specific biomarkers or advanced testing could improve early identification, especially in women, who often present with a more subtle clinical profile compared to men [11,12,13]

From a therapeutic perspective, while HFrEF benefits from well-established treatments, pharmacological options for HFpEF remain more limited. However, recent studies such as EMPEROR-PRESERVED and DELIVER have introduced sodium–glucose cotransporter 2 (SGLT2) inhibitors, which have shown the potential to modify disease progression and prognosis [14,15,16].

Gender differences in treatment response represent an active area of research, aiming to develop personalized approaches to improve prognosis and quality of life for patients.

This review will explore sex differences in the pathophysiology of diastolic dysfunction, the progression to HFpEF, diagnostic and therapeutic challenges, as well as future research perspectives in this field.

## 2. Definition, Epidemiology, and Pathophysiological Mechanisms

Heart failure with preserved ejection (HFPEF) is a clinical syndrome in which the heart is unable to deliver the requisite amount of oxygen to the tissues commensurate with their metabolic needs or does so but only at the expense of increased left ventricular filling pressures, despite a normal ejection fraction. The prevalence of HFPEF is increasing, due to a greater awareness of the diagnosis and refined echocardiographic techniques and also due to changes in demographics (such as ageing of the population) and higher burden of lifestyle-related risk factors (such as obesity and diabetes) [17,18,19].

The current American Heart Association/American College of Cardiology and European Society of Cardiology guidelines both recommend that a diagnosis of HFPEF should be based on the presence of the three following features: 1. signs and symptoms consistent with a diagnosis of heart failure; 2. absence of depressed ejection fraction (i.e., a left ventricular ejection fraction [LVEF] ≥ 50%); and 3. objective measures showing a diastolic dysfunction [20,21]

Diastolic dysfunction refers to abnormal mechanical properties of the myocardium and includes abnormal left ventricular (LV) diastolic distensibility, impaired filling, and slow or delayed relaxation, regardless of whether patient is asymptomatic or symptomatic and whether or not the EF is normal or depressed. Diastolic dysfunction implies that the myofibrils do not rapidly or completely return to their resting length, the ventricle cannot accept blood at low pressures, and ventricular filling is slow or incomplete unless atrial pressure rises [22].

Important sex/gender differences have been described in HF subtypes. Although the incidence of HFpEF is similar among men and women, the prevalence of HFpEF is higher in women compared with men. In one study, women out-numbered men 2:1 with respect to HFpEF hospitalizations. This is further reflected in lifetime risk estimates of HF: the lifetime risk of HFpEF is nearly double that of HFrEF among women (10.7% vs. 5.8%), whereas the lifetime risk of HFpEF and HFrEF are similar among men [23]. Studies on ethnic differences in HFpEF burden are evolving. Data from the ARIC (Atherosclerosis Risk In Communities) study of HF-related hospitalizations in four U.S. communities between 2005 and 2014 show that average event rates for the first HFpEF hospitalization were highest among Black women (7.4 per 1000 person-years [95% CI: 6.7–8.1 per 1000 person-years]) when compared with Black men (6.2 per 1000 person-years [95% CI: 5.5–7.0 per 1000 person-years]), White women (5.9 per 1000 person-years [95% CI: 5.5–6.2 per 1000 person-years]), and White men (4.9 per 1000 person-years [95% CI: 4.5–5.3 per 1000 person-years]). During this time period, the annual percent change for the first HFpEF hospitalization increased for all four race–sex groups and was particularly pronounced among Black women [24].

Although the exact mechanisms underlying sex-specific differences in HFpEF prevalence remain incompletely understood, several hypotheses have emerged. Potential contributing factors include enhanced inflammatory responses and chronic microvascular dysfunction, which may be more pronounced in women and are thought to significantly contribute to the development and progression of HFpEF [25,26].

Additionally, classical cardiovascular risk factors such as diabetes mellitus, obesity, hypertension, and coronary artery disease appear to disproportionately influence HFpEF risk in women. Recent evidence also highlights the relevance of gender-specific risk factors, particularly early-onset menopause and adverse pregnancy outcomes, including polycystic ovary syndrome, pre-eclampsia, gestational diabetes, and recurrent pregnancy losses. These factors are increasingly recognized for their potential role in predisposing women to HFpEF. The increased prevalence of HFpEF among older women, particularly during postmenopause, has led researchers to examine the role of estradiol deficiency as a critical contributor to disease pathogenesis. Estradiol modulates several pathways integral to cardiovascular health, including inflammation regulation, oxidative stress management, endothelial function, and nitric oxide signaling. Consequently, the menopausal decline in estradiol is associated with adverse changes in body composition, blood pressure regulation, and lipid metabolism, factors that collectively heighten cardiovascular risk and contribute to the pathophysiology of HFpEF [27,28,29]

HFpEF is recognized as a syndrome characterized by notable phenotypic diversity, influenced significantly by sex-based differences in clinical presentation and disease progression. Women typically exhibit more pronounced concentric left ventricular remodeling and severe diastolic impairment compared to men, reflecting intrinsic differences in myocardial and vascular adaptation to underlying risk factors [26,30].

Age has also emerged as a critical determinant in defining HFpEF phenotypes, with younger patients often presenting as obese men, while older patients frequently are women with a higher burden of cardiovascular and metabolic comorbidities [31].

Recent advances in phenotyping methodologies allow for a more precise characterization of distinct patient subgroups within the HFpEF population. For instance, a recent analysis utilizing advanced phenotyping identified three discrete groups in a cohort of 397 HFpEF patients: younger women presenting with less severe ventricular remodeling, predominantly obese and diabetic women, and older patients of both genders who exhibited pronounced chronic kidney disease and significant adverse left ventricular remodeling [32].

**Figure 1:**
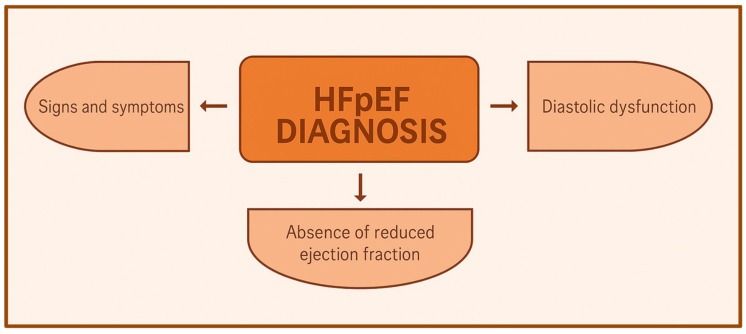
Risk factors and disease progression.

Furthermore, notable sex-related differences extend beyond disease prevalence and clinical profiles to include outcomes and patient experiences. Specifically, while women with HFpEF generally report lower quality of life compared to men, paradoxically, their overall survival rates tend to be superior (Figure 1) [33,34].

Traditional HF risk factors increase susceptibility for HFpEF, particularly age, blood pressure, obesity, and diabetes mellitus.

Age is a main risk factor, since with aging the heart loses elasticity due to myocardial fibrosis, increasing the prevalence of diastolic dysfunction, which can reach up to 50% in the elderly [35].

Arterial hypertension is also a major trigger for diastolic dysfunction, as it increases the workload on the heart, causing left ventricular hypertrophy and reducing the heart’s ability to relax, leading to greater ventricular stiffness [36].

Obesity contributes to hypertension, metabolic syndrome, and insulin resistance, leading to a state of systemic inflammation, endothelial dysfunction, and subsequent myocyte remodeling, favoring myocardial stiffness and fibrosis [37,38,39]

Diabetes mellitus with chronic hyperglycemia and insulin resistance can cause advanced glycosylation of cardiac proteins, promoting fibrosis and stiffness of the left ventricle [40]. It also contributes to HFpEF pathogenesis via systemic vascular inflammation. The production of reactive oxygen species and decreased bioavailability of nitric oxide leads to downstream lowering of soluble guanylate cyclase and protein kinase G activity in cardiomyocytes. Deficient PKG activity subsequently impairs myocardial relaxation and induces cardiomyocyte hypertrophy [41].

There is a growing recognition of the concept of risk factor clustering, particularly during the menopausal transition and postmenopause. It is crucial to consider that life expectancy has increased, and in Western countries, women are expected to spend approximately 40% of their lives in a state of postmenopause. This extended phase of life has significant implications for cardiovascular health and the development of HFpEF. HFpEF has a higher prevalence in women compared to men. This sex-based discrepancy appears to be driven by differences in cardiovascular adaptation to comorbidities and distinct underlying etiologic mechanisms [35,41].

Women, especially after menopause, are more predisposed to HFpEF, with a higher prevalence than men, especially in the elderly. Some common risk factors in women are more pronounced, increasing the risk of developing and worsening HFpEF [30].

In fact, in postmenopausal women, isolated systolic hypertension is prevalent, favoring ventricular stiffness, concentric hypertrophy, and progression to HFpEF. There are also intrinsic physiological characteristics in women that predispose them to HFpEF, including increased arterial stiffness [42].

In contrast, men with HFpEF tend to have greater LV mass and volume, lower arterial elastance, and a higher incidence of coronary artery disease, indicating a hemodynamically distinct pathophysiological substrate compared to women [30].

Hormonal differences also play a crucial role: postmenopausal estrogen reduction increases the risk of fibrosis and ventricular stiffness. Estrogens modulate inflammatory and fibrotic pathways, and their decline in women accelerates the progression of diastolic dysfunction, whereas such modulation is absent in men. In addition, women with HFpEF respond less to drug treatments than men, with a lower therapeutic response to ACE inhibitors, angiotensin II antagonists, and beta-blockers, probably due to biological differences [43].

Furthermore, women tend to develop a more obese phenotype, with a higher incidence of metabolic syndrome and diabetes mellitus and worse diastolic dysfunction. Conversely, in men, visceral adiposity is more closely linked to epicardial fat accumulation and myocardial lipid infiltration, which may influence diastolic function differently [44].

In conclusion, women, especially in postmenopause, are more vulnerable to diastolic dysfunction, with a more rapid progression to HFpEF. This progression is often driven by hormonal, vascular, and metabolic alterations that differ from the ischemic and structural remodeling mechanisms more typical in men. These differences require greater understanding to develop personalized treatments and targeted preventive strategies for men and women.

There is a strong impact of some comorbidities with the worsening of diastolic dysfunction leading to HFpEF, in particular atrial fibrillation (AF) and renal dysfunction.

Paroxysmal and permanent atrial fibrillation is common in HFpEF because the two conditions share common risk factors and because of the impairment of ventricular filling during diastole and the reduced filling volume, worsening diastolic dysfunction [45].

AF in HFpEF patients predicts an unfavorable prognosis, with a rapid deterioration of cardiac function and an increased risk of hospitalizations and mortality [46].

This association can also be frequent because chronic, sustained, or intermittent elevation in left ventricular (LV) filling pressures in HFpEF cause remodeling and dysfunction of the left atrium over time, frequently culminating in the development of AF, which may be considered as a “biomarker” of underlying LA myopathy and an indicator of more advanced HFpEF. Furthermore, patients with permanent AF are more congested with greater pulmonary vascular disease and lower cardiac output. In conclusion, survival decreased with increasing AF burden [45].

Also, renal dysfunction is usually common in patients with HFpEF and affects the progression of diastolic dysfunction. Chronic renal failure and reduced renal perfusion are often found in these patients, aggravated by high filling pressure and sodium retention. Renal dysfunction increases myocardial fibrosis and left ventricular hypertrophy, worsening the rigidity of the left ventricle and its ability to relax during diastole [47]. In addition, the reduced ability to eliminate fluids causes pulmonary congestion and central venous pressure, further exacerbating diastolic dysfunction.

So, both AF and renal dysfunction contribute to increased ventricular stiffness and myocardial fibrosis, reducing the elastance of the heart, compromising its ability to expand during diastole, and increasing filling pressure. In addition, fluid retention in patients with renal dysfunction worsens venous congestion and increases the load on the heart, accentuating diastolic dysfunction.

The presence of these comorbidities in patients with HFpEF is a negative prognostic indicator, accelerating the progression of diastolic dysfunction and worsening clinical outcomes. Properly managing these conditions is essential to improve prognosis. The treatment of AF, with rhythm control and prevention of arrhythmic episodes, and the management of renal dysfunction, which includes diuresis therapy and blood pressure control, are essential to improve diastolic function and prevent worsening heart failure.

## 3. Pathogenesis and Triggering Factors

Systemic arterial hypertension is a frequent disease and is among the most frequent cardiovascular risk factors. It plays an important pathogenetic role in a lot of cardiac diseases. Half of the patients with hypertension develop diastolic dysfunction. This condition is characterized by a persistent increased afterload that leads to stretching of the myocytes and an increase in the concentrations of angiotensin II in vascular and myocardial tissue which is known to be one of the main factors responsible for “genetic reprogramming.” [48,49,50].

It stimulates cells to grow and the production of the extracellular matrix, leading to increased myocardial mass and to an increased LV stiffness. LVDD is also caused by metabolic alterations: in some studies, it has been found that chronic hypertension also affects myocardial HEP metabolism, resulting in impaired diastolic function [51].

Another important clinical condition related to diastolic dysfunction is obesity. Obesity is a very frequent condition nowadays, especially in western societies. This condition has been demonstrated to be closely linked to the development of diastolic dysfunction; indeed, visceral obesity is related to diastolic dysfunction: it is responsible for concentric LV remodeling, elevated myocardial triglyceride levels, and impaired energetics. In some studies, it has also been shown that metabolic mechanisms may be more important than structural factors in mediating the negative effects of obesity on diastolic function [52,53].

Diabetes mellitus is a very frequent disease that carries a high risk of cardiovascular complications and death. Diabetes mellitus leads to macrovascular and microvascular complication, and it is one of the major cardiovascular risk factors for coronary artery disease. However, diabetes is also associated with diastolic dysfunction, due to different etiologies: abnormalities in high-energy phosphate metabolism, impaired calcium transport, interstitial accumulation of advanced glycosylation end products, imbalance in collagen synthesis and degradation, abnormal microvascular function, activation of the cardiac renin–angiotensin system, decreased adiponectin levels, and alteration in the metabolism of free fatty acids and glucose [54].

In a cross-sectional study that included 1778 participants (70% males and 30% females), it has been demonstrated that in female patients affected by type II diabetes, the development of left ventricular diastolic dysfunction was more frequent [55].

Gestational diabetes mellitus (GDM) represents an additional important pathogenetic condition linked to future cardiovascular complications. Recent evidence demonstrates that GDM independently predicts the occurrence of subclinical myocardial dysfunction in the early postpartum period and significantly increases the risk for HFpEF years after delivery [56,57].

Systemic autoimmune diseases have also been related to diastolic dysfunction, likely secondary to chronic inflammatory states. There have been a lot of studies demonstrating a relation between autoimmune syndrome and LVDD.

In a study of 244 patients with rheumatoid arthritis, they discovered a higher prevalence of diastolic dysfunction, which was independently associated with the disease duration and IL-6 levels, demonstrating the impact of chronic autoimmune diseases in myocardial function [58].

Systemic sclerosis is another important autoimmune syndrome associated with left ventricle diastolic dysfunction. In a study of 225 patients with systemic sclerosis, it has been shown that LVDD is a common finding in this disease that progresses over time and is associated with more severe dyspnea, even though it is not always associated with HfpEF, but it can explain symptoms [59].

The menopausal transition and postmenopausal syndrome are additional important conditions related with diastolic dysfunction. There is an exponential increase in the incidence of heart failure with preserved ejection fraction compared with men of the same age in postmenopausal woman, which might indicate a potential role of hormonal changes in subclinical and clinical diastolic dysfunction. Estrogen deficiency leads to impaired intracellular calcium metabolism by the receptor ERa subtypes ERa, ERb, and GPR30 which act as inhibitors of myocardial hypertrophy. Estrogens also increase diastolic function by modulating natriuretic peptides. These hormones also inhibit the proliferation of fibroblasts, leading to reduced myocardial fibrosis and myocardial stiffness, which are both causes of left ventricular diastolic dysfunction [60,61].

Cardiac amyloidosis represents another important clinical condition associated with diastolic dysfunction. It is an infiltrative cardiomyopathy characterized by two main phenotypes, secondary to the deposition of different proteins: (AL-CM) immunoglobulin light chain amyloidosis and (ATTR-CM) transthyretin amyloidosis. The deposition of these proteins leads to myocardial hypertrophy (often with low voltages on ECG) and severe diastolic dysfunction, which can progress to a completely impaired diastolic function and a restrictive phenotype.

ATTR-CM, particularly the wild-type form (ATTRwt), has been predominantly diagnosed in older men, with studies indicating a male-to-female ratio of approximately 7:1. However, this discrepancy may be influenced by underdiagnosis in women, who often present at an older age with less pronounced left ventricular hypertrophy and a higher ejection fraction. Additionally, certain hereditary variants (ATTRv), such as the Val122Ile mutation, may affect women at a higher rate than previously recognized. AL amyloidosis does not exhibit a clear sex predilection but is often underdiagnosed in women due to atypical presentations [62,63].

**Figure 2:**
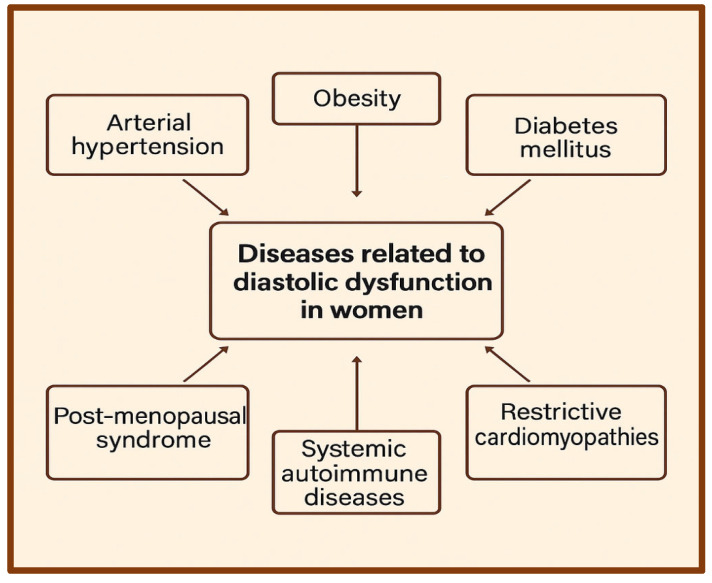
Diagnostic approaches and differential diagnosis.

More broadly, various diseases contribute to the risk and pathophysiology of diastolic dysfunction, with sex-specific risk factors influencing disease manifestation and progression. The underrepresentation of women in many clinical studies has led to an underestimation of the true burden of diastolic dysfunction in females. Traditional cardiovascular risk factors such as hypertension, diabetes, and obesity appear to have a more significant impact on LV diastolic dysfunction (LVDD) in women. This may explain, in part, why women with HFpEF experience higher hospitalization rates, worse quality of life, and poorer prognosis compared to men [64]. However, further studies are needed to clarify sex-specific differences in amyloidosis prevalence, presentation, and therapeutic approaches (Figure 2).

Diastolic dysfunction is a key pathophysiological feature of HFpEF and is associated with increased cardiovascular morbidity and mortality [65]. Evaluating diastolic function relies primarily on echocardiographic parameters, which provide essential hemodynamic and structural insights into left ventricular relaxation, stiffness, and filling pressures. Echocardiographic assessment is based on mitral inflow Doppler, tissue Doppler imaging (TDI), pulmonary venous flow, and left atrial (LA) volume measurements. The mitral inflow Doppler pattern, obtained from pulsed-wave Doppler, assesses early (E) and late (A) diastolic filling velocities, their ratio (E/A), and the deceleration time (DT). A reduced E/A ratio (<0.8) and prolonged DT indicate impaired relaxation, while restrictive patterns suggest increased LV filling pressures [6].

TDI, which measures myocardial velocities at the mitral annulus, is crucial for differentiating normal aging from pathological dysfunction. The E/e′ ratio, calculated as mitral inflow E velocity divided by e′, estimates LV filling pressures. An E/e′ > 14 indicates elevated pressures, while values of 8–14 require further correlation [66].

Another essential parameter is left atrial volume index (LAVI), a chronic marker of elevated LV filling pressures. Increased LAVI (>34 mL/m^2^) is associated with adverse outcomes, including atrial fibrillation and HFpEF [67]. Pulmonary venous Doppler flow and LA strain analysis further aid in assessing diastolic dysfunction progression [6].

In addition to conventional Doppler and TDI techniques, advanced echocardiographic tools enhance the evaluation of diastolic dysfunction by providing more precise myocardial function characterization.

Speckle-tracking echocardiography (STE) assesses myocardial strain to evaluate subtle impairments in LV relaxation and LA function. Global longitudinal strain (GLS) reduction, even in patients with normal ejection fraction, is an early marker of subclinical diastolic dysfunction [68].

LA strain and LA ejection fraction (LAEF) have gained significant attention in diastolic function assessment. LA strain, particularly LA reservoir strain, reflects atrial compliance and is reduced in the presence of increased LV filling pressures. Sex-specific differences have been recognized, with women demonstrating slightly higher normative values of LA reservoir strain compared to men; however, thresholds for pathological strain reduction are typically uniform (<23%) but may require further refinement in future guidelines [69]. Similarly, LAEF, calculated as (LA maximum volume − LA minimum volume)/LA maximum volume, provides insight into LA contractile function and is an independent predictor of cardiovascular outcomes in HFpEF [70].

Although current guidelines do not specify sex-specific cut-offs for LAEF, ongoing research suggests potential variability that should be considered in future diagnostic criteria.

Three-dimensional (3D) echocardiography provides more accurate volumetric measurements of LA volume and LV mass, refining risk stratification for patients with suspected HFpEF [71].

Sex-specific reference ranges and cut-offs for key echocardiographic and MRI-based parameters are outlined in Table 1, underscoring the importance of sex-specific diagnostic criteria to enhance accuracy

However, resting echocardiographic assessment may not always detect early diastolic dysfunction, particularly in HFpEF. Exercise stress echocardiography is a valuable tool for unmasking diastolic dysfunction in borderline cases. This test evaluates changes in the E/e′ ratio, tricuspid regurgitation velocity, and pulmonary artery pressures under exertion. A stress-induced E/e′ > 15 suggests impaired diastolic reserves, characteristic of latent HFpEF [72,73,74]

In addition to echocardiography, cardiac magnetic resonance (CMR) imaging with T1 mapping enables precise myocardial characterization, particularly in detecting fibrosis and interstitial expansion, key contributors to diastolic dysfunction. Extracellular volume fraction (ECV) quantification on CMR correlates with diastolic dysfunction severity and clinical outcomes [75].

**Table 1:** Sex-specific echocardiographic and cardiac magnetic resonance (CMR) parameters for the diagnosis and risk stratification of diastolic dysfunction and HFpEF. The table summarizes reference values for women and men, highlighting subtle but clinically relevant differences in left atrial reservoir strain, left atrial volume index (LAVI), global longitudinal strain (GLS), and extracellular volume fraction (ECV). These distinctions underscore the need for sex-adapted diagnostic thresholds to improve the accuracy of HFpEF identification.

Parameter	Women	Men	Bibliography
LA Reservoir Strain (%)	≥35 (normal), <23 (abnormal)	≥ 33 (normal), <23 (abnormal)	[6,69]
LAVI (mL/m^2^)	<34 (normal), ≥34 (abnormal)	<34 (normal), ≥34 (abnormal)	[67]
GLS (%)	≤−20 (normal), >−16 (abnormal)	≤−19 (normal), >−15 (abnormal)	[68]
ECV (%)	<28 (normal), ≥28 (abnormal)	<27 (normal), ≥27 (abnormal)	[75]

Recent data suggest that women may exhibit greater extracellular volume expansion despite similar levels of fibrosis on histology, which could influence ECV thresholds for clinical interpretation. Sex-specific normal values for T1 and ECV mapping are therefore increasingly advocated.

Biomarkers, both established and emerging, also play a critical role in the diagnosis and management of HFpEF, particularly in differentiating HFpEF from other conditions with similar presentations. Established biomarkers include natriuretic peptides such as BNP and NT-proBNP, with sex-specific reference ranges recognized to account for physiological differences—women typically present with slightly higher baseline values. Emerging biomarkers such as Galectin-3, ST2, and markers of collagen turnover have also shown promise, with ongoing research required to refine sex-specific diagnostic thresholds and therapeutic implications [76,77,78,79]

Epidemiologic studies reveal that diastolic dysfunction prevalence varies significantly based on age and sex. Population-based studies indicate that diastolic dysfunction is highly prevalent, affecting up to 25–30% of individuals over 65 years old, with prevalence increasing with age [66].

Sex differences are particularly evident, with women—especially when postmenopausal—exhibiting a higher prevalence of HFpEF than men, despite similar or even lower levels of traditional cardiovascular risk factors [25]. The higher prevalence in women has been attributed to sex-specific myocardial adaptations, including greater LV stiffness, smaller LV cavity size, and higher arterial elastance, which contribute to increased susceptibility to diastolic dysfunction despite preserved ejection fraction [80,81,82]

Moreover, these adaptations may influence diagnostic accuracy and call for tailored algorithms: for instance, reliance on absolute LA size or wall thickness without indexing to body surface area can underestimate pathology in women.

One major challenge is the underrepresentation of women in clinical trials investigating HFpEF and diastolic function, leading to gaps in understanding sex-specific pathophysiology and response to therapies [83].

## 4. Therapeutic Strategies and Outcomes

Until recently, there was limited robust evidence supporting the prognostic benefits of a specific pharmacological treatment for heart failure with preserved ejection fraction (HFpEF), a condition primarily driven by diastolic dysfunction. The latest ESC heart failure guidelines reported a class I recommendation exclusively for patients with signs and symptoms of congestion [84].

Several clinical trials including ACE-I/ARB and ARNI have not demonstrated a significant improvement in their respective primary endpoints in this patient setting.

In the TOP-CAT trial, including patients with heart failure and EF > 45%, spironolactone, although it did not improve the primary end point compared to placebo, had demonstrated a significant reduction in hospitalization for heart failure alone.

In the PARAGON-HF trial, which included 4822 patients with heart failure with EF > 45% and in NYHA class II-IV, ARNI was not shown to significantly reduce the primary endpoint (mortality from cardiovascular causes or hospitalization for heart failure), compared to valsartan alone [85].

However, recent randomized clinical trials have demonstrated the efficacy of certain therapies in improving patient outcomes.

Sodium–glucose cotransporter-2 inhibitors (SGLT2i), originally developed for diabetes management, have shown significant cardiovascular benefits in patients with HFpEF.

The EMPEROR-PRESERVED trial demonstrated that treatment with empagliflozin (10 mg/day) led to a 21% relative risk reduction in cardiovascular mortality and heart failure hospitalization compared to placebo not only in patients with HFrEF but also with an ejection fraction > 40%, over a median follow-up of 26 months. In addition to the ejection fraction, other study inclusion criteria were an NYHA functional class equal to or greater than 2 and NT-proBNP values greater than 300 pg/mL in the case of a sinus rhythm or 900 pg/mL in the case of patients with an atrial fibrillation rhythm [16].

Similarly, the DELIVER trial, including 6223 patient with heart failure and an ejection fraction > 40%, found that dapagliflozin (10 mg/day) reduced the relative risk of the same composite endpoint by 18% over a median follow-up of 2.3 years [15].

Unlike the EMPEROR-PRESERVED trial, the DELIVER trial also included patients with a previous reduced ejection fraction, which subsequently became > 40%.

An emerging therapeutic option is represented by glucagon-like peptide-1 receptor agonists (GLP-1 RAs). The SUMMIT trial, including 731 patients with heart failure, an ejection fraction > 50%, and obesity (BMI > 30) reported that treatment with tirzepatide (administered subcutaneously up to 15 mg/week) led to a 38% relative risk reduction in cardiovascular mortality and worsening heart failure over a follow-up period of approximately 100 weeks. Tirzepatide is the first drug shown to improve cardiovascular outcomes in patients with heart failure and an ejection fraction of no less than 50% [86].

These findings suggest that therapies originally designed for glycemic control in diabetic patients may also confer significant cardiovascular benefits, regardless of ejection fraction.

Sex differences in treatment response remains an active area of research. Several studies suggest that biological sex may influence pharmacokinetics, myocardial stiffness, neurohormonal activation, and metabolic reserve, thereby affecting therapeutic efficacy in HFpEF. Some studies suggest that women may derive greater benefit from SGLT2i therapy compared to men, potentially due to differences in ventricular stiffness and cardiac reserve mechanisms. However, further research is needed to elucidate these effects and to develop more targeted therapeutic strategies. In particular, subgroup analyses from major trials have begun to shed light on sex-specific responses. In the EMPEROR-PRESERVED trial, women experienced a 25% relative risk reduction in the primary outcome compared to 19% in men, although this difference did not reach statistical significance. Similarly, in the DELIVER trial, the relative risk reduction was 23% in women versus 15% in men. These findings, although exploratory, raise the hypothesis that the cardioprotective effects of SGLT2i may be more pronounced in women—possibly due to differences in LV compliance, preload sensitivity, or natriuretic peptide regulation [15,16].

The SUMMIT trial also provided notable data: among the 731 patients enrolled, women represented 53% of the population and showed a consistent benefit in both quality of life and clinical outcomes when treated with tirzepatide, again suggesting possible sex-specific advantages that merit further exploration. Preclinical data suggest that GLP-1 RAs may exert more potent metabolic and anti-inflammatory effects in females, which could partly explain the observed clinical differences. Nevertheless, dedicated mechanistic studies are needed (Table 2) [86].

First of all, the female sex is well represented and constitutes approximately 44% of all patients recruited in the EMPEROR-PRESERVED and DELIVER HF trials and 53% of patients recruited in the Summit trial. Furthermore, the prognostic benefit was also demonstrated in this subgroup of patients in all trials, with a more favorable trend, although it was not statistically significant compared to the male sex. Specifically, in the EMPEROR-PRESERVED trial, there was a 25% relative risk reduction in the primary outcome (mortality from cardiovascular causes and hospitalization for heart failure), while in the DELIVER HF trial there was a 19% relative risk reduction in the same subgroup with similar primary outcome [15,16,86].

Despite these findings, sex-stratified analysis remains underpowered in most studies, and none of the current guidelines provide sex-specific recommendations. Future trials should include prespecified sex-based subgroup analyses to better guide personalized HFpEF management.

Non-pharmacological interventions, including lifestyle optimization and cardiac rehabilitation, also play a crucial role in HFpEF management. Regular physical activity and isotonic exercise has been associated with improved functional capacity and quality of life, with some evidence suggesting greater benefits in women, possibly due to higher baseline impairment in physical performance. However, adherence to these programs remains a significant challenge, necessitating targeted interventions to enhance accessibility and patient engagement.

In conclusion, while the treatment of HFpEF has historically been challenging, emerging evidence strongly supports the use of SGLT2i and GLP-1 RAs as effective strategies for improving clinical outcomes. Nevertheless, key questions remain regarding the optimization of therapy based on sex-specific and aging differences and the integration of pharmacological and non-pharmacological approaches for more effective disease management.

## 5. Knowledge Gaps and Future Directions

In recent years, significant progress has been made in understanding left ventricular diastolic dysfunction (LVDD) and HFpEF as distinct entities. However, substantial gaps remain regarding the mechanisms underlying the progression from LVDD to HFpEF, which are hypothesized to be sex-specific. Established risk factors such as hypertension, diabetes, and obesity appear to play a particularly relevant role in women. However, additional risk factors that are either more prevalent or unique to the female population, including those associated with pregnancy, may be underrecognized and require further investigation [64].

HFpEF is a heterogeneous syndrome involving multiple pathophysiological abnormalities, making it challenging to apply a uniform therapeutic approach to all patients. Categorizing patients based on clinical and pathophysiological phenotypes represents a crucial step toward personalized treatment strategies. Echocardiography plays an essential role in the assessment of HFpEF, providing valuable insights into disease pathophysiology, phenotyping, and prognosis. However, noninvasive diagnosis remains complex in the absence of fluid retention and overt congestion. In patients presenting with exertional dyspnea, objective documentation of elevated left ventricular filling pressures—whether invasively or noninvasively—is necessary. The integration of clinical characteristics with echocardiographic findings can support diagnosis and guide decision-making regarding advanced testing [87,88,89]

Current knowledge gaps include the precise definition of HFpEF phenotypes, the role of noninvasive imaging and biomarkers in risk stratification, and the application of advanced technologies, such as artificial intelligence, to refine diagnostic capabilities. Standardizing diagnostic criteria is crucial for early disease identification, enabling targeted therapies and improving overall HFpEF management [64].

## 6. Conclusions

Diastolic dysfunction and heart failure with preserved ejection fraction (HFpEF) represent an increasing clinical challenge, with a particularly high prevalence among women. Gender differences in pathophysiology, diagnosis, and treatment response highlight the need for personalized approaches in both clinical management and research.

From a pathophysiological perspective, women exhibit greater ventricular stiffness and heightened susceptibility to risk factors such as hypertension, obesity, and diabetes, with menopause playing a key role in disease progression. However, current diagnostic criteria may not be sufficiently sensitive to these specificities, emphasizing the need for broader implementation of advanced tools such as strain imaging and cardiac magnetic resonance.

Therapeutic strategies for HFpEF remain limited compared to heart failure with reduced ejection fraction (HFrEF), but recent studies have demonstrated the efficacy of SGLT2 inhibitors in improving clinical outcomes. Considering gender differences in treatment response is crucial to optimizing therapy and enhancing patients’ quality of life.

In the future, a deeper understanding of gender-specific characteristics in diastolic dysfunction will enable the development of targeted therapeutic strategies, reducing the gender gap and improving the prognosis of this growing patient population.

## Figures and Tables

**Table 2:** Outcomes of major randomized clinical trials in patients with HFpEF, stratified by sex. The table reports the total number of participants, the proportion of female participants, and the relative risk reduction (RRR) for heart failure events in women and men. In the EMPEROR-PRESERVED and DELIVER trials, SGLT2 inhibitors demonstrated comparable efficacy across sexes, with a trend toward greater benefit in women. In the SUMMIT trial, which evaluated the GLP-1 receptor agonist tirzepatide, a 38% reduction in heart failure events was observed in both sexes, with women representing over 50% of the study population. These findings highlight the relevance of sex-specific subgroup analyses in assessing therapeutic efficacy in HFpEF.

TRIAL	Total Participants	Female Participants (%)	RRR in Women	RRR in Men
**EMPEROR-** **Preserved**	5.988	2.676 (44.7%)	HR 0.75	HR 0.81
**DELIVER**	6.263	2.747 (43.9%)	HR 0.81	HR 0.82
**SUMMIT**	731	388 (53%)	38% reduction in HF events	38% reduction in HF events

## References

[1] Gazewood J.D., Turner P.L. (2017). Heart Failure with Preserved Ejection Fraction: Diagnosis and Management. Am. Fam. Physician.

[2] Rame J.E., Ramilo M., Spencer N., Blewett C., Mehta S.K., Dries D.L., Drazner M.H. (2004). Development of a depressed left ventricular ejection fraction in patients with left ventricular hypertrophy and a normal ejection fraction. Am. J. Cardiol..

[3] Pfeffer M.A., Shah A.M., Borlaug B.A. (2019). Heart Failure with Preserved Ejection Fraction In Perspective. Circ. Res..

[4] Kass D.A., Bronzwaer J.G.F., Paulus W.J. (2004). What mechanisms underlie diastolic dysfunction in heart failure?. Circ. Res..

[5] Kitabatake A., Inoue M., Asao M., Tanouchi J., Masuyama T., Abe H., Morita H., Senda S., Matsuo H. (1982). Transmitral blood flow reflecting diastolic behavior of the left ventricle in health and disease--a study by pulsed Doppler technique. Jpn. Circ. J..

[6] Nagueh S.F., Smiseth O.A., Appleton C.P., Byrd B.F., Dokainish H., Edvardsen T., Flachskampf F.A., Gillebert T.C., Klein A.L., Lancellotti P. (2016). Recommendations for the Evaluation of Left Ventricular Diastolic Function by Echocardiography: An Update from the American Society of Echocardiography and the European Association of Cardiovascular Imaging. J. Am. Soc. Echocardiogr..

[7] Andersen O.S., Smiseth O.A., Dokainish H., Abudiab M.M., Schutt R.C., Kumar A., Sato K., Harb S., Gude E., Remme E.W. (2017). Estimating Left Ventricular Filling Pressure by Echocardiography. J. Am. Coll. Cardiol..

[8] Lancellotti P., Galderisi M., Edvardsen T., Donal E., Goliasch G., Cardim N., Magne J., Laginha S., Hagendorff A., Haland T.F. (2017). Echo-Doppler estimation of left ventricular filling pressure: Results of the multicentre EACVI Euro-Filling study. Eur. Heart, J. Cardiovasc. Imaging.

[9] Lee M.P., Glynn R.J., Schneeweiss S., Lin K.J., Patorno E., Barberio J., Levin R., Evers T., Wang S.V., Desai R.J. (2020). Risk Factors for Heart Failure with Preserved or Reduced Ejection Fraction Among Medicare Beneficiaries: Application of Competing Risks Analysis and Gradient Boosted Model. Clin. Epidemiol..

[10] Mattioli A.V., Coppi F., Migaldi M., Farinetti A. (2018). Physical activity in premenopausal women with asymptomatic peripheral arterial disease. J. Cardiovasc. Med..

[11] Anker S.D., Usman M.S., Anker M.S., Butler J., Böhm M., Abraham W.T., Adamo M., Chopra V.K., Cicoira M., Cosentino F. (2023). Patient phenotype profiling in heart failure with preserved ejection fraction to guide therapeutic decision making. A scientific statement of the Heart Failure Association, the European Heart Rhythm Association of the European Society of Cardiology, and the European Society of Hypertension. Eur. J. Heart Fail..

[12] Wohlfahrt P., Redfield M.M., Lopez-Jimenez F., Melenovsky V., Kane G.C., Rodeheffer R.J., Borlaug B.A. (2014). Impact of general and central adiposity on ventricular-arterial aging in women and men. JACC Heart Fail..

[13] Mattioli A.V., Moscucci F., Sciomer S., Maffei S., Nasi M., Pinti M., Bucciarelli V., Dei Cas A., Parati G., Ciccone M.M. (2023). Cardiovascular prevention in women: An update by the Italian Society of Cardiology working group on «Prevention, hypertension and peripheral disease». J. Cardiovasc. Med..

[14] Rist A., Sevre K., Wachtell K., Devereux R.B., Aurigemma G.P., Smiseth O.A., Kjeldsen S.E., Julius S., Pitt B., Burnier M. (2024). The current best drug treatment for hypertensive heart failure with preserved ejection fraction. Eur. J. Intern. Med..

[15] Solomon S.D., McMurray J.J.V., Claggett B., de Boer R.A., DeMets D., Hernandez A.F., Inzucchi S.E., Kosiborod M.N., Lam C.S.P., Martinez F. (2022). Dapagliflozin in Heart Failure with Mildly Reduced or Preserved Ejection Fraction. N. Engl. J. Med..

[16] Anker S.D., Butler J., Filippatos G., Ferreiram J.P., Bocchi E., Böhm M., Brunner-La Rocca H.P., Choi D.J., Chopra V., Chuquiure-Valenzuela E. (2021). Empagliflozin in Heart Failure with a Preserved Ejection Fraction. N. Engl. J. Med..

[17] Cleland J.G.F., Pellicori P. (2013). Defining diastolic heart failure and identifying effective therapies. JAMA.

[18] Vasan R.S., Levy D. (2000). Defining diastolic heart failure: A call for standardized diagnostic criteria. Circulation.

[19] Mattioli A.V., Coppi F., Nasi M., Pinti M., Gallina S. (2023). Long COVID: A New Challenge for Prevention of Obesity in Women. Am. J. Lifestyle Med..

[20] McMurray J.J., Adamopoulos S., Anker S.D., Auricchio A., Böhm M., Dickstein K., Falk V., Filippatos G., Fonseca C., Gomez-Sanchez M.A. (2012). ESC Guidelines for the diagnosis and treatment of acute and chronic heart failure 2012: The Task Force for the Diagnosis and Treatment of Acute and Chronic Heart Failure 2012 of the European Society of Cardiology. Developed in collaboration with the Heart Failure Association (HFA) of the ESC. Eur. Heart J..

[21] Yancy C.W., Jessup M., Bozkurt B., Butler J., Casey D.E., Drazner M.H., Fonarow G.C., Geracim S.A., Horwich T., Januzzi J.L. (2013). 2013 ACCF/AHA guideline for the management of heart failure: A report of the American College of Cardiology Foundation/American Heart Association Task Force on practice guidelines. Circulation.

[22] Gaasch W.H., Zile M.R. (2004). Left ventricular diastolic dysfunction and diastolic heart failure. Annu. Rev. Med..

[23] Chang P.P., Wruck L.M., Shahar E., Rossi J.S., Loehr L.R., Russell S.D., Agarwal S.K., Konety S.H., Rodriguez C.J., Rosamond W.D. (2018). Trends in Hospitalizations and Survival of Acute Decompensated Heart Failure in Four US Communities (2005–2014): ARIC Study Community Surveillance. Circulation.

[24] Shah S.J., Lam C.S.P., Svedlund S., Saraste A., Hage C., Tan R.S., Beussink-Nelson L., Ljung Faxén U., Fermer M.L., Broberg M.A. (2018). Prevalence and correlates of coronary microvascular dysfunction in heart failure with preserved ejection fraction: PROMIS-HFpEF. Eur. Heart J..

[25] Beale A.L., Meyer P., Marwick T.H., Lam C.S.P., Kaye D.M. (2018). Sex Differences in Cardiovascular Pathophysiology: Why Women Are Overrepresented in Heart Failure with Preserved Ejection Fraction. Circulation.

[26] Beale A.L., Nanayakkara S., Segan L., Mariani J.A., Maeder M.T., van Empel V., Vizi D., Evans S., Lam C.S.P., Kaye D.M. (2019). Sex Differences in Heart Failure with Preserved Ejection Fraction Pathophysiology: A Detailed Invasive Hemodynamic and Echocardiographic Analysis. JACC Heart Fail..

[27] Gökçe M., Karahan B., Erdöl C., Kasap H., Ozdemirci S. (2003). Left ventricular diastolic function assessment by tissue Doppler echocardiography in relation to hormonal replacement therapy in postmenopausal women with diastolic dysfunction. Am. J. Ther..

[28] Coppi F., Cavalletti A., Pagnoni G., Campani C., Grossule F., Maini A., Macripò P., Zanini G., Sinigaglia G., Giuggioli D. (2025). Pulmonary hypertension in patients with Sjögren’s syndrome: Gender differences in cardiovascular risk factors and instrumental data. Int. J. Cardiol..

[29] Sabbatini A.R., Kararigas G. (2020). Menopause-Related Estrogen Decrease and the Pathogenesis of HFpEF: JACC Review Topic of the Week. J. Am. Coll. Cardiol..

[30] Sotomi Y., Hikoso S., Nakatani D., Mizuno H., Okada K., Dohi T., Kitamura T., Sunaga A., Kida H., Oeun B. (2021). Sex Differences in Heart Failure with Preserved Ejection Fraction. J. Am. Heart Assoc..

[31] Tromp J., Shen L., Jhund P.S., Anand I.S., Carson P.E., Desai A.S., Granger C.B., Komajda M., McKelvie R.S., Pfeffer M.A. (2019). Age-Related Characteristics and Outcomes of Patients with Heart Failure with Preserved Ejection Fraction. J. Am. Coll. Cardiol..

[32] Shah S.J., Katz D.H., Selvaraj S., Burke M.A., Yancy C.W., Gheorghiade M., Bonow R.O., Huang C.C., Deo R.C. (2015). Phenomapping for novel classification of heart failure with preserved ejection fraction. Circulation.

[33] Lewis E.F., Lamas G.A., O’Meara E., Granger C.B., Dunlap M.E., McKelvie R.S., Probstfield J.L., Young J.B., Michelson E.L., Halling K. (2007). Characterization of health-related quality of life in heart failure patients with preserved versus low ejection fraction in CHARM. Eur. J. Heart Fail..

[34] Goyal P., Paul T., Almarzooq Z.I., Peterson J.C., Krishnan U., Swaminathan R.V., Feldman D.N., Wells M.T., Karas M.G., Sobol I. (2017). Sex- and Race-Related Differences in Characteristics and Outcomes of Hospitalizations for Heart Failure with Preserved Ejection Fraction. J. Am. Heart Assoc..

[35] Ponikowski P., Voors A.A., Anker S.D., Bueno H., Cleland J.G.F., Coats A.J.S., Falk V., González-Juanatey J.R., Harjola V.P., Jankowska E.A. (2016). 2016 ESC Guidelines for the diagnosis and treatment of acute and chronic heart failure: The Task Force for the diagnosis and treatment of acute and chronic heart failure of the European Society of Cardiology (ESC)Developed with the special contribution of the Heart Failure Association (HFA) of the ESC. Eur. Heart J..

[36] Zile M.R., Simsic J.M. (2000). Diastolic heart failure: Diagnosis and treatment. Clin. Cornerstone.

[37] Lavie C.J., Milani R.V., Ventura H.O. (2009). Obesity and cardiovascular disease: Risk factor, paradox, and impact of weight loss. J. Am. Coll. Cardiol..

[38] Rossi R., Talarico M., Schepis F., Coppi F., Sgura F.A., Monopoli D.E., Minici R., Boriani G. (2021). Effects of sildenafil on right ventricle remodelling in Portopulmonary hypertension. Pulm. Pharmacol. Ther..

[39] Borlaug B.A., Sharma K., Shah S.J., Ho J.E. (2023). Heart Failure with Preserved Ejection Fraction: JACC Scientific Statement. J. Am. Coll. Cardiol..

[40] From A.M., Scott C.G., Chen H.H. (2010). The Development of Heart Failure in Patients with Diabetes Mellitus and Preclinical Diastolic Dysfunction: A Population Based Study. J. Am. Coll. Cardiol..

[41] Kaur G., Lau E. (2022). Sex differences in heart failure with preserved ejection fraction: From traditional risk factors to sex-specific risk factors. Womens Health.

[42] Kannel W.B. (2007). Hypertensive Risk Assessment: Cardiovascular Risk Factors and Hypertension. J. Clin. Hypertens..

[43] Redfield M.M. (2016). Heart Failure with Preserved Ejection Fraction. N. Engl. J. Med..

[44] Alpert M.A., Lavie C.J., Agrawal H., Aggarwal K.B., Kumar S.A. (2014). Obesity and heart failure: Epidemiology, pathophysiology, clinical manifestations, and management. Transl. Res..

[45] Reddy Y.N.V., Obokata M., Verbrugge F.H., Lin G., Borlaug B.A. (2020). Atrial Dysfunction in Patients with Heart Failure with Preserved Ejection Fraction and Atrial Fibrillation. J. Am. Coll. Cardiol..

[46] Fauchier L., Bisson A., Bodin A. (2023). Heart failure with preserved ejection fraction and atrial fibrillation: Recent advances and open questions. BMC Med..

[47] Patel R.N., Sharma A., Prasad A., Bansal S. (2023). Heart Failure with Preserved Ejection Fraction with CKD: A Narrative Review of a Multispecialty Disorder. Kidney Med..

[48] Sadoshima J., Xu Y., Slayter H.S., Izumo S. (1993). Autocrine release of angiotensin II mediates stretch-induced hypertrophy of cardiac myocytes in vitro. Cell.

[49] Mattioli A.V., Coppi F., Bucciarelli V., Gallina S. (2023). Cardiovascular risk stratification in young women: The pivotal role of pregnancy. J. Cardiovasc. Med..

[50] Van Heugten H.A., De Jonge H.W., Bezstarosti K., Sharma H.S., Verdouw P.D., Lamers J.M. (1995). Intracellular signaling and genetic reprogramming during agonist-induced hypertrophy of cardiomyocytes. Ann. N. Y. Acad. Sci..

[51] Lamb H.J., Beyerbacht H.P., van der Laarse A., Stoel B.C., Doornbos J., van der Wall E.E., de Roos A. (1999). Diastolic dysfunction in hypertensive heart disease is associated with altered myocardial metabolism. Circulation.

[52] Lortet S., Heckmann M., Aussedat J., Ray A., Vincent M., Sassard J., Zimmer H.G., Rossi A. (1993). Alteration of cardiac energy state during development of hypertension in rats of the Lyon strain: A 31P-NMR study on the isolated rat heart. Acta Physiol. Scand..

[53] Savonitto G., Barbisan D., Ameri P., Lombardi C.M., Driussi M., Gentile P., Howard L., Toma M., Pagnesi M., Collini V. (2025). Characteristics, Prognosis and ESC/ERS Risk Stratification in Obese Patients with Pulmonary Arterial Hypertension (PAH). Chest.

[54] Tsujino T., Kawasaki D., Masuyama T. (2006). Left ventricular diastolic dysfunction in diabetic patients: Pathophysiology and therapeutic implications. Am. J. Cardiovasc. Drugs.

[55] Wang Y., Zhou Y., Zhang Y., Ren Q., Wang Y., Su H. (2023). Female is Associated with Left Ventricular Diastolic Dysfunction in Patients with Type 2 Diabetes. Diabetes Metab. Syndr. Obes..

[56] Sonaglioni A., Bordoni T., Naselli A., Nicolosi G.L., Grasso E., Bianchi S., Ferrulli A., Lombardo M., Ambrosio G. (2024). Influence of gestational diabetes mellitus on subclinical myocardial dysfunction during pregnancy: A systematic review and meta-analysis. Eur. J. Obstet. Gynecol. Reprod. Biol..

[57] Pabón M.A., Misra A., Honigberg M.C. (2023). Adverse pregnancy outcomes and future risk of heart failure. Curr. Opin. Cardiol..

[58] Liang K.P., Myasoedova E., Crowson C.S., Davis J.M., Roger V.L., Karon B.L., Borgeson D.D., Therneau T.M., Rodeheffer R.J., Gabriel S.E. (2010). Increased prevalence of diastolic dysfunction in rheumatoid arthritis. Ann. Rheum. Dis..

[59] Ross L., Patel S., Stevens W., Burns A., Prior D., La Gerche A., Nikpour M. (2022). The clinical implications of left ventricular diastolic dysfunction in systemic sclerosis. Clin. Exp. Rheumatol..

[60] Maslov P.Z., Kim J.K., Argulian E., Ahmadi A., Narula N., Singh M., Bax J., Narula J. (2019). Is Cardiac Diastolic Dysfunction a Part of Post-Menopausal Syndrome?. JACC Heart Fail..

[61] Delcuratolo E., Palazzuoli A., Coppi F., Mattioli A.V., Severino P., Tramonte F., Fedele F. (2023). Risk Factors and Cellular Differences in Heart Failure: The Key Role of Sex Hormones. Biomedicines.

[62] Vilches S., Martínez-Avial M., Méndez I., Gómez González C., Espinosa M.Á. (2024). Sex Differences in Transthyretin Cardiac Amyloidosis: Unraveling the Complexities in Epidemiology, Pathophysiology, Diagnosis, and Treatment. Curr. Heart Fail. Rep..

[63] Arno S., Cowger J. (2022). The genetics of cardiac amyloidosis. Heart Fail. Rev..

[64] van Ommen A.M.L.N., Canto E.D., Cramer M.J., Rutten F.H., Onland-Moret N.C., den Ruijter H.M. (2022). Diastolic dysfunction and sex-specific progression to HFpEF: Current gaps in knowledge and future directions. BMC Med..

[65] Paulus W.J., Tschöpe C., Sanderson J.E., Rusconi C., Flachskampf F.A., Rademakers F.E., Marino P., Smiseth O.A., De Keulenaer G., Leite-Moreira A.F. (2007). How to diagnose diastolic heart failure: A consensus statement on the diagnosis of heart failure with normal left ventricular ejection fraction by the Heart Failure and Echocardiography Associations of the European Society of Cardiology. Eur. Heart, J..

[66] Redfield M.M., Jacobsen S.J., Burnett J.C., Mahoney D.W., Bailey K.R., Rodeheffer R.J. (2003). Burden of systolic and diastolic ventricular dysfunction in the community: Appreciating the scope of the heart failure epidemic. JAMA.

[67] Tsang T.S.M., Barnes M.E., Gersh B.J., Bailey K.R., Seward J.B. (2002). Left atrial volume as a morphophysiologic expression of left ventricular diastolic dysfunction and relation to cardiovascular risk burden. Am. J. Cardiol..

[68] DeVore A.D., McNulty S., Alenezi F., Ersboll M., Vader J.M., Oh J.K., Lin G., Redfield M.M., Lewis G., Semigran M.J. (2017). Impaired left ventricular global longitudinal strain in patients with heart failure with preserved ejection fraction: Insights from the RELAX trial. Eur. J. Heart Fail..

[69] Inoue K., Khan F.H., Remme E.W., Ohte N., García-Izquierdo E., Chetrit M., Moñivas-Palomero V., Mingo-Santos S., Andersen Ø.S., Gude E. (2021). Determinants of left atrial reservoir and pump strain and use of atrial strain for evaluation of left ventricular filling pressure. Eur. Heart, J. Cardiovasc. Imaging.

[70] Melenovsky V., Hwang S.J., Redfield M.M., Zakeri R., Lin G., Borlaug B.A. (2015). Left atrial remodeling and function in advanced heart failure with preserved or reduced ejection fraction. Circ. Heart Fail..

[71] Henein M.Y., Lindqvist P. (2020). Diastolic function assessment by echocardiography: A practical manual for clinical use and future applications. Echocardiography.

[72] Borlaug B.A., Nishimura R.A., Sorajja P., Lam C.S.P., Redfield M.M. (2010). Exercise hemodynamics enhance diagnosis of early heart failure with preserved ejection fraction. Circ. Heart Fail..

[73] Coppi F., Pagnoni G., Campani C., Grossule F., Vacchi C., Giuggioli D., Mattioli A.V., Boriani G. (2025). Sjögren’s syndrome and pulmonary hypertension: Exploring the intricate link with interstitial lung disease. Int. J. Cardiol..

[74] de Pinto M., Coppi F., Spinella A., Pagnoni G., Morgante V., Macripò P., Boschini M., Guerra A.F., Tampieri F., Secchi O. (2024). The predictive role of the TAPSE/sPAP ratio for cardiovascular events and mortality in systemic sclerosis with pulmonary hypertension. Front. Cardiovasc. Med..

[75] Rommel K.P., von Roeder M., Latuscynski K., Oberueck C., Blazek S., Fengler K., Besler C., Sandri M., Lücke C., Gutberlet M. (2016). Extracellular Volume Fraction for Characterization of Patients with Heart Failure and Preserved Ejection Fraction. J. Am. Coll. Cardiol..

[76] Daniels L.B., Maisel A.S. (2007). Natriuretic peptides. J. Am. Coll. Cardiol..

[77] de Boer R.A., Voors A.A., Muntendam P., van Gilst W.H., van Veldhuisen D.J. (2009). Galectin-3: A novel mediator of heart failure development and progression. Eur. J. Heart Fail..

[78] de Boer R.A., Daniels L.B., Maisel A.S., Januzzi J.L. (2015). State of the Art: Newer biomarkers in heart failure. Eur. J. Heart Fail..

[79] Bayes-Genis A., de Antonio M., Galán A., Sanz H., Urrutia A., Cabanes R., Cano L., González B., Díez C., Pascual T. (2012). Combined use of high-sensitivity ST2 and NTproBNP to improve the prediction of death in heart failure. Eur. J. Heart Fail..

[80] Gori M., Lam C.S., Gupta D.K., Santos A.B., Cheng S., Shah A.M., Claggett B., Zile M.R., Kraigher-Krainer E., Pieske B. (2014). Sex-specific cardiovascular structure and function in heart failure with preserved ejection fraction. Eur. J. Heart Fail..

[81] Colaci M., Giuggioli D., Manfredi A., Sebastiani M., Coppi F., Rossi R., Ferri C. (2012). Aortic pulse wave velocity measurement in systemic sclerosis patients. Reumatismo.

[82] Regitz-Zagrosek V., Kararigas G. (2017). Mechanistic Pathways of Sex Differences in Cardiovascular Disease. Physiol. Rev..

[83] Shah S.J., Katz D.H., Deo R.C. (2014). Phenotypic spectrum of heart failure with preserved ejection fraction. Heart Fail. Clin..

[84] McDonagh T.A., Metra M., Adamo M., Gardner R.S., Baumbach A., Böhm M., Burri H., Butler J., Čelutkienė J., Chioncel O. (2021). 2021 ESC Guidelines for the diagnosis and treatment of acute and chronic heart failure. Eur. Heart J..

[85] Solomon S.D., McMurray J.J.V., Anand I.S., Ge J., Lam C.S.P., Maggioni A.P., Martinez F., Packer M., Pfeffer M.A., Pieske B. (2019). Angiotensin-Neprilysin Inhibition in Heart Failure with Preserved Ejection Fraction. N. Engl. J. Med..

[86] Packer M., Zile M.R., Kramer C.M., Baum S.J., Litwin S.E., Menon V., Ge J., Weerakkody G.J., Ou Y., Bunck M.C. (2025). Tirzepatide for Heart Failure with Preserved Ejection Fraction and Obesity. N. Engl. J. Med..

[87] Zhang J., Gajjala S., Agrawal P., Tison G.H., Hallock L.A., Beussink-Nelson L., Lassen M.H., Fan E., Aras M.A., Jordan C.M. (2018). Fully Automated Echocardiogram Interpretation in Clinical Practice. Circulation.

[88] Rossi R., Coppi F., Monopoli D.E., Sgura F.A., Arrotti S., Boriani G. (2022). Pulmonary arterial hypertension and right ventricular systolic dysfunction in COVID-19 survivors. Cardiol. J..

[89] Obokata M., Reddy Y.N.V., Borlaug B.A. (2020). Diastolic Dysfunction and Heart Failure with Preserved Ejection Fraction: Understanding Mechanisms by Using Noninvasive Methods. JACC Cardiovasc. Imaging.

